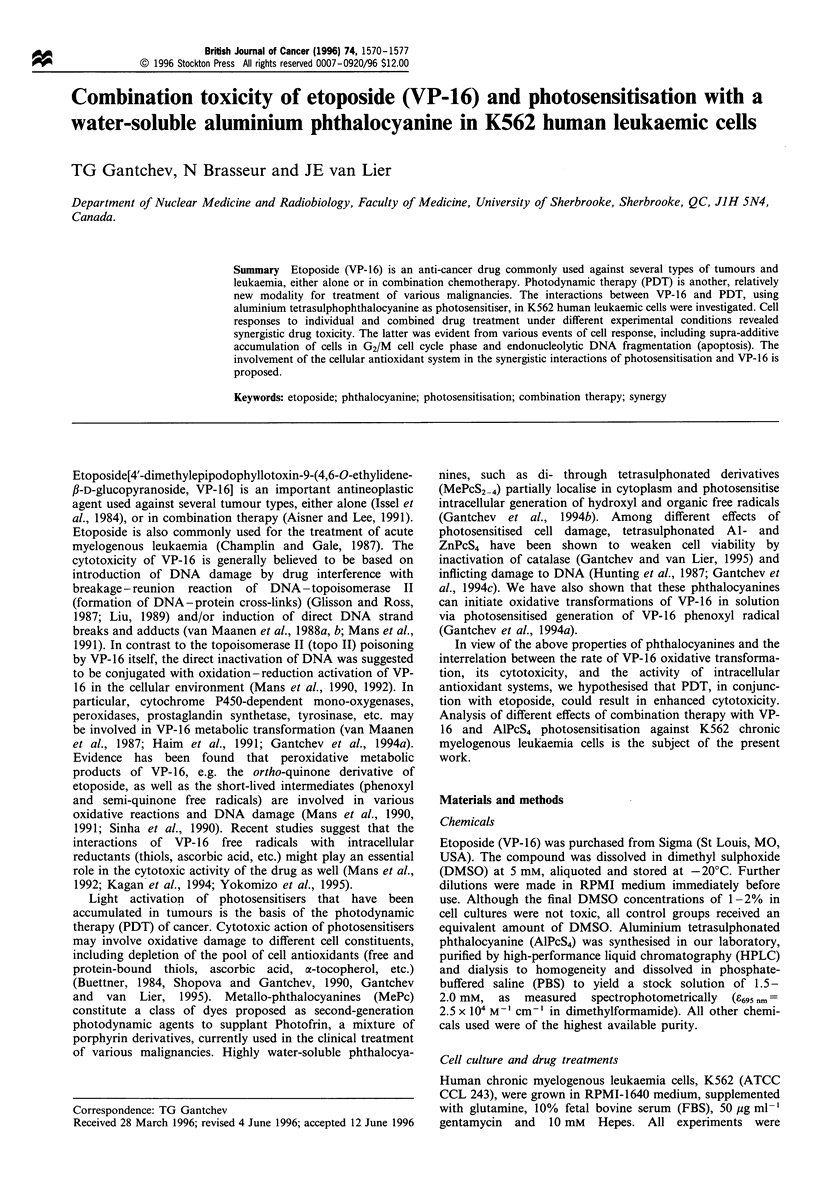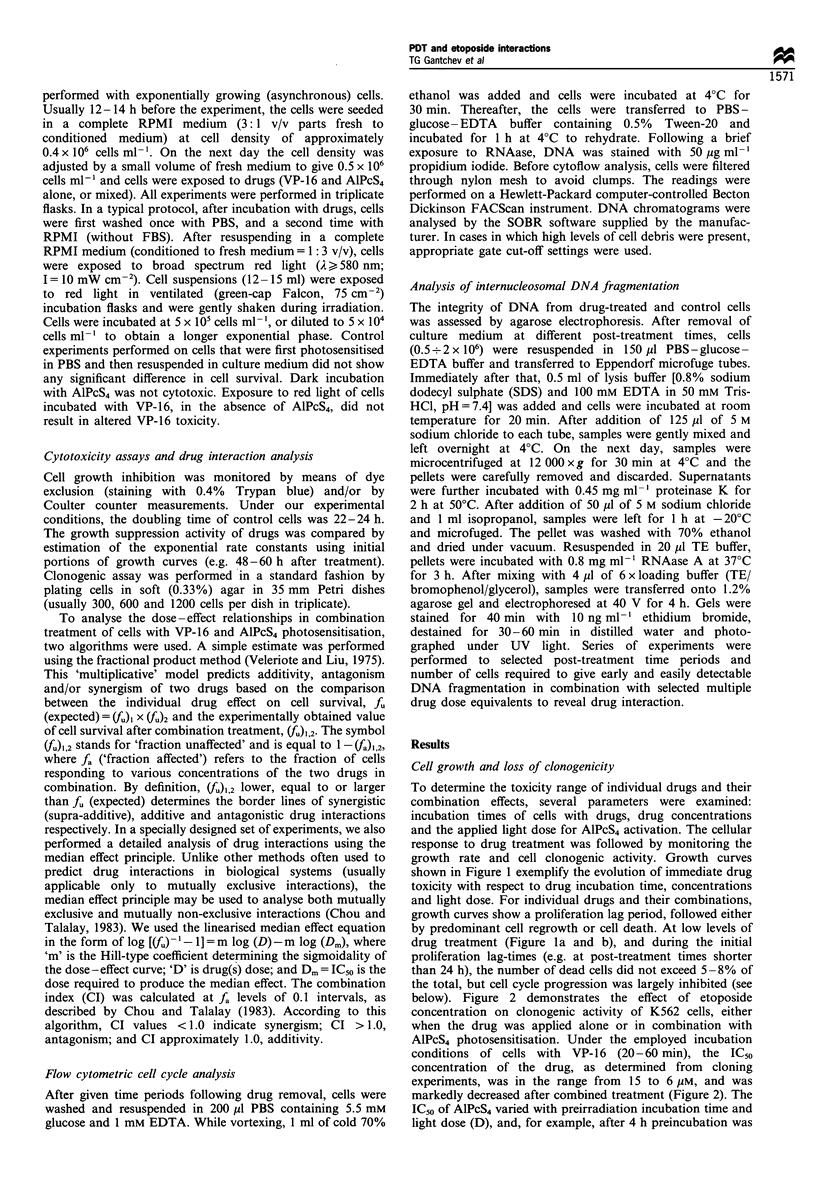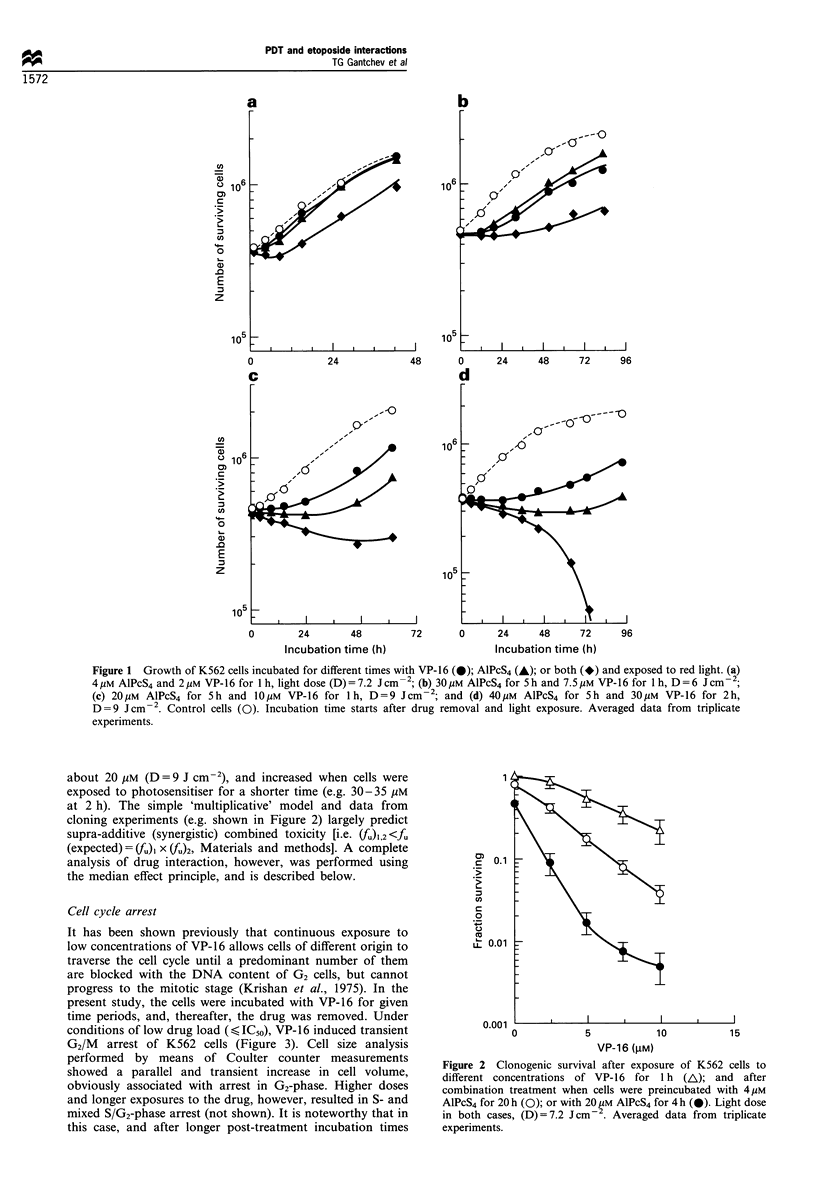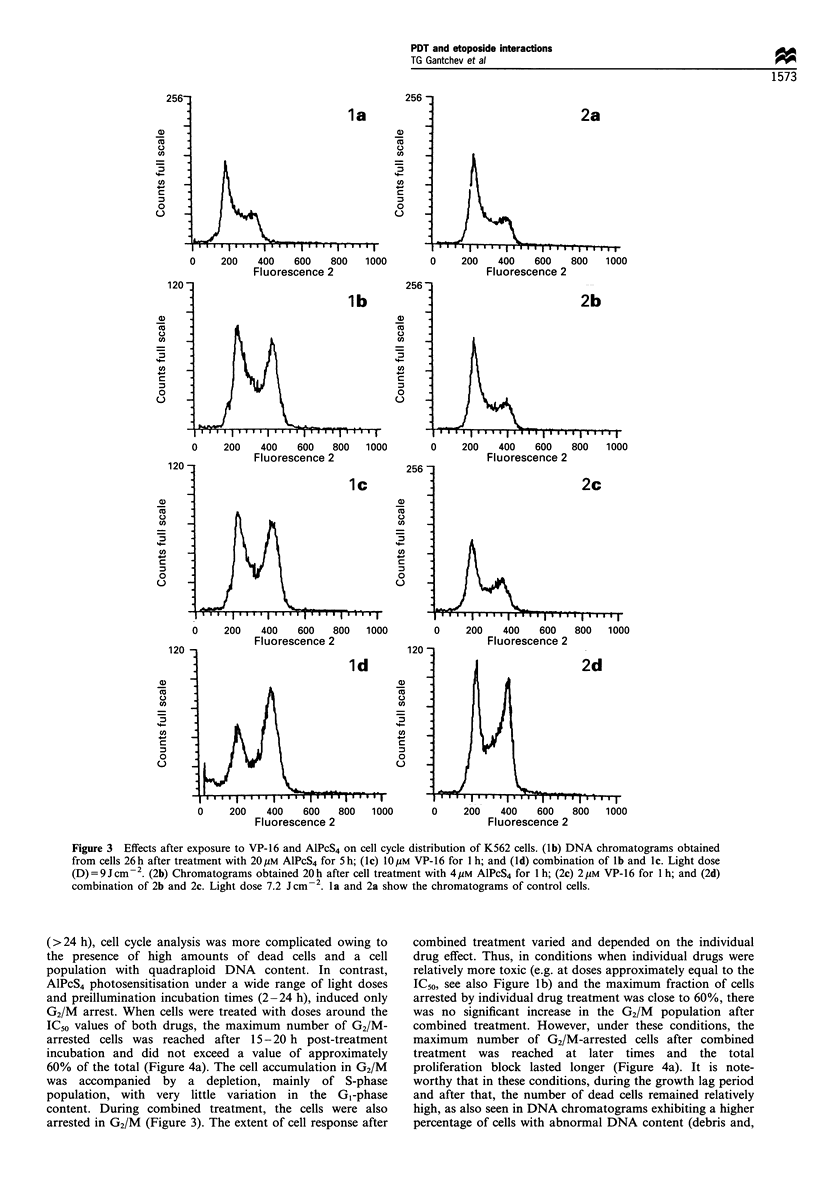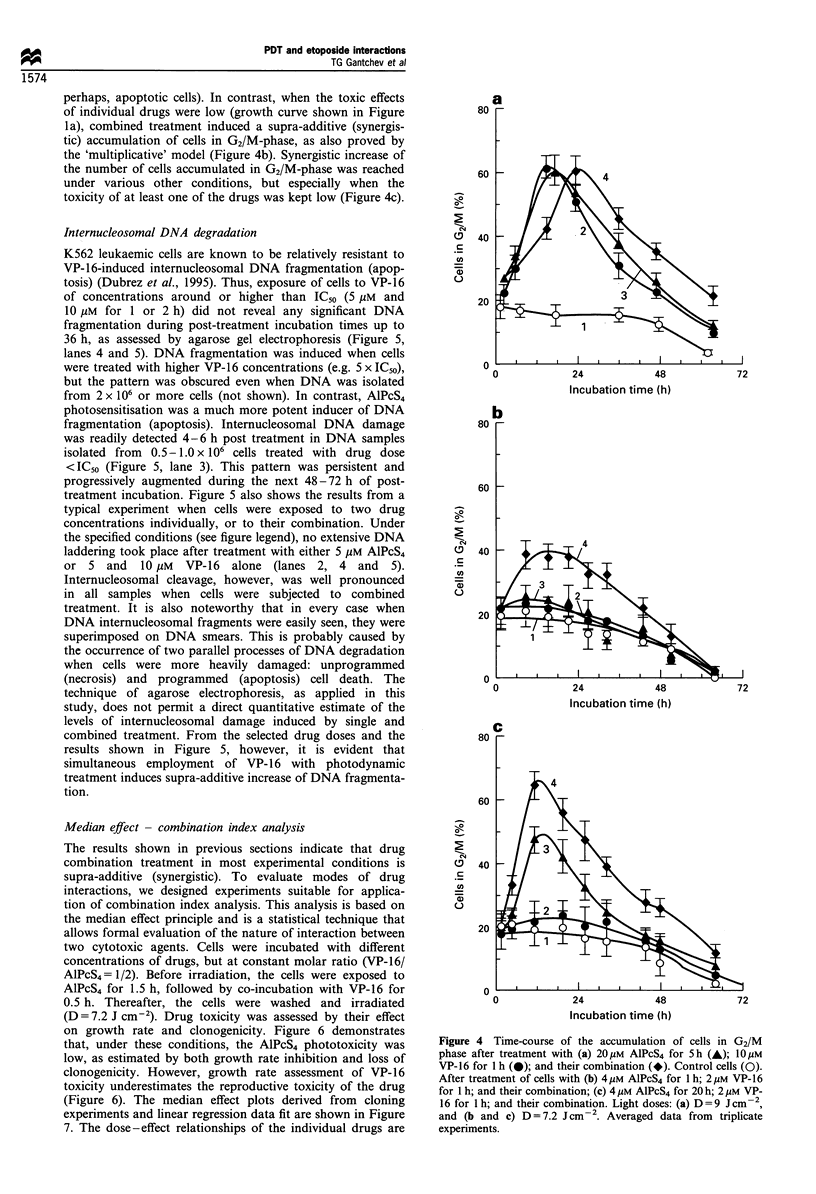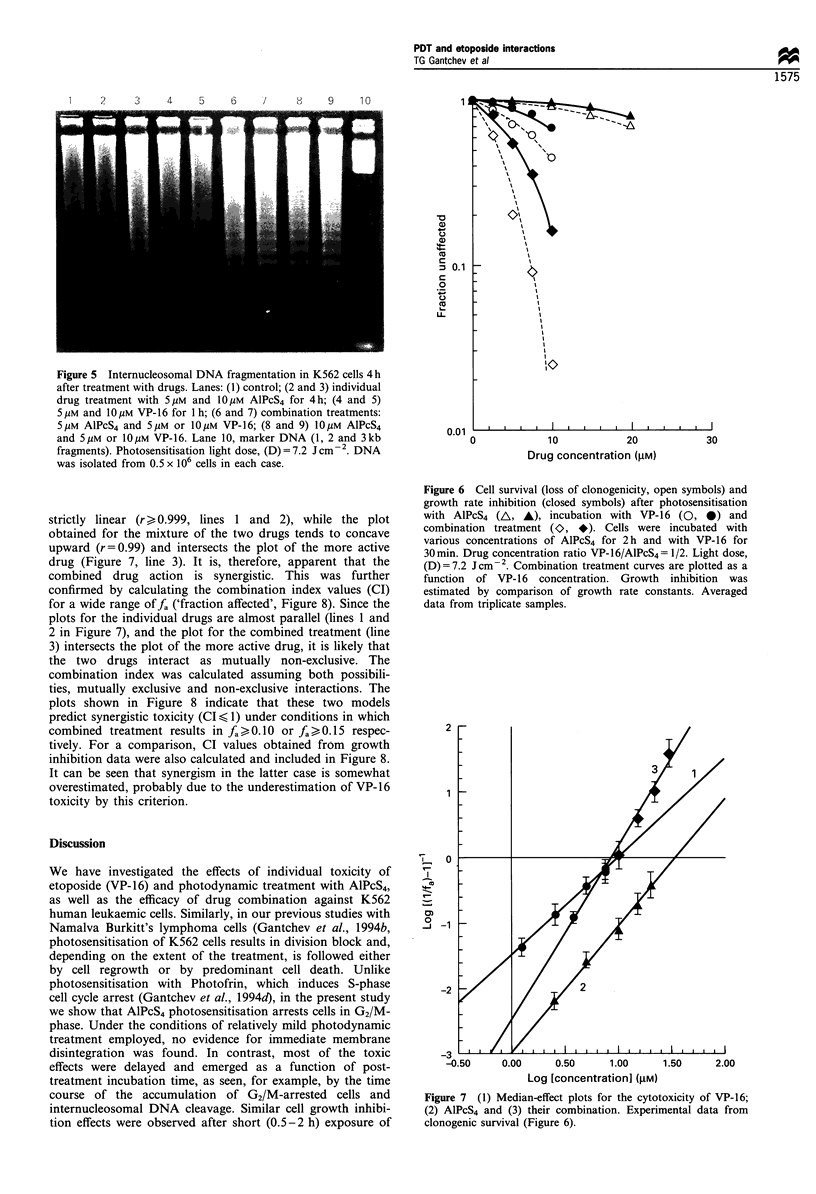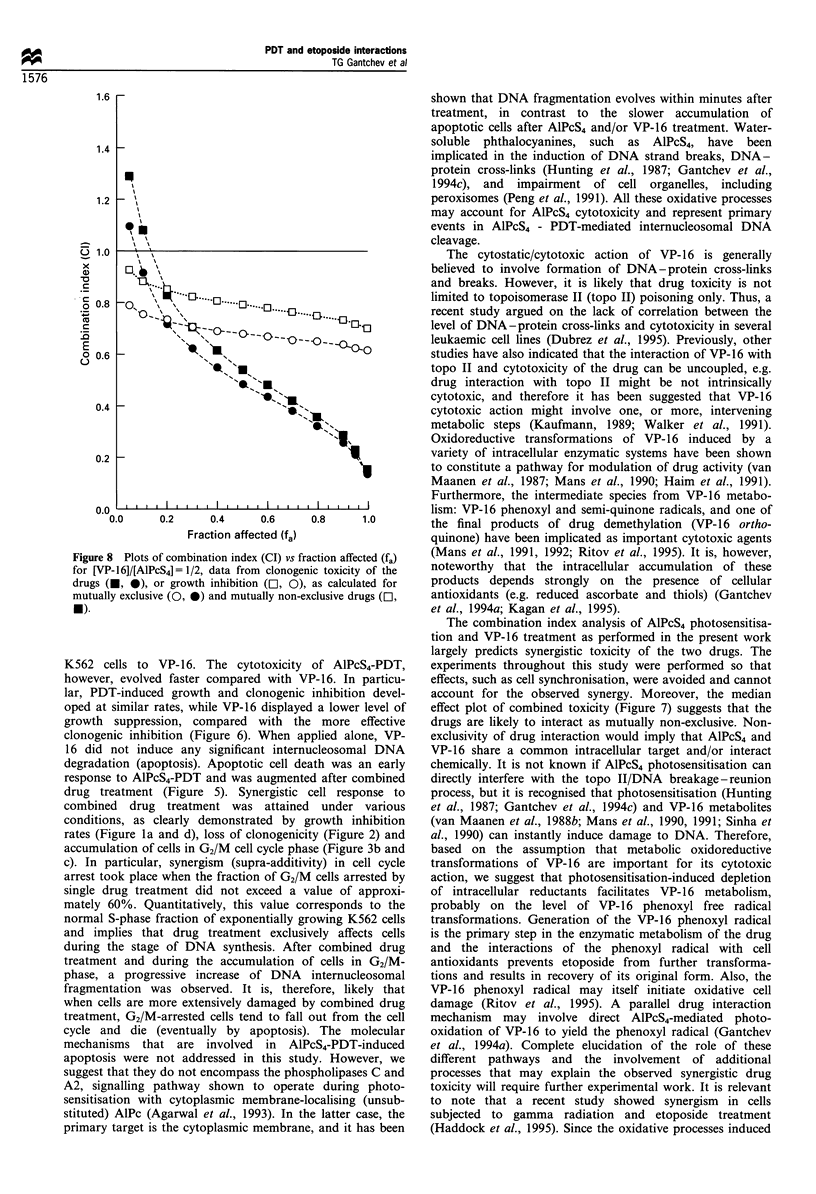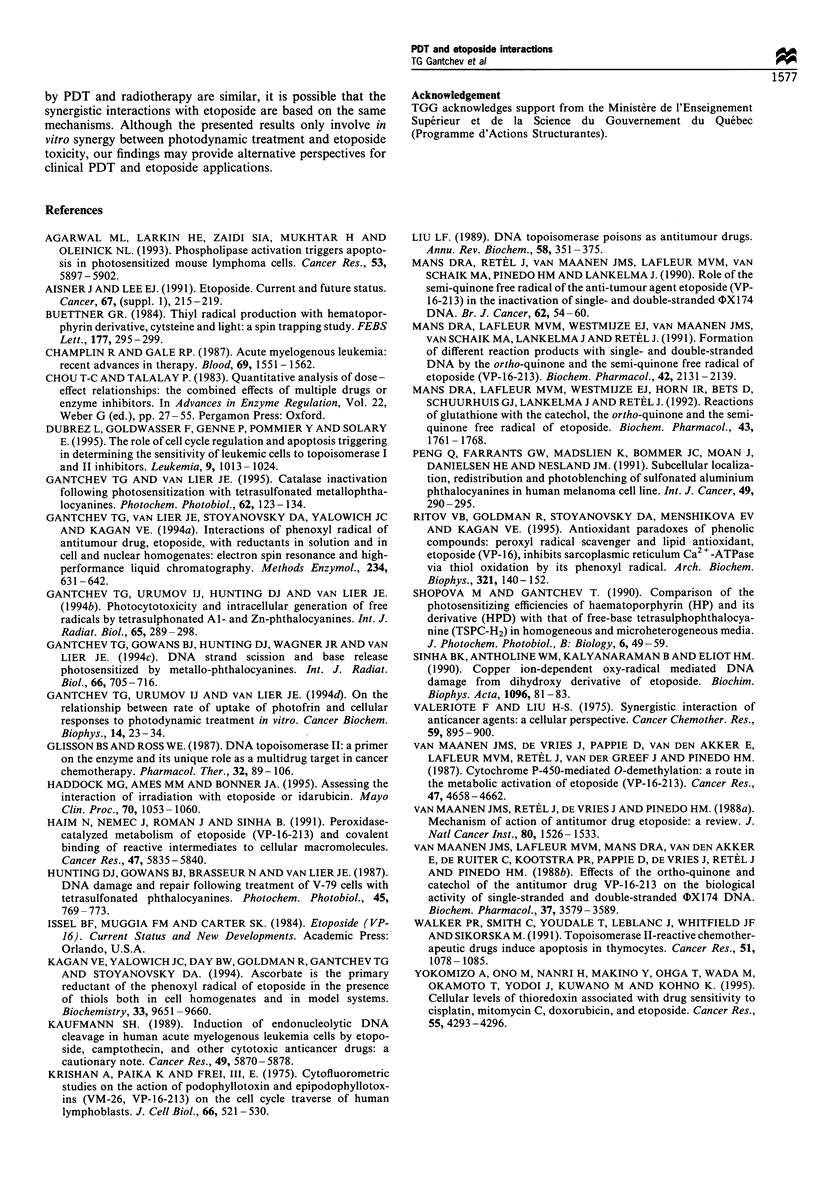# Combination toxicity of etoposide (VP-16) and photosensitisation with a water-soluble aluminium phthalocyanine in K562 human leukaemic cells.

**DOI:** 10.1038/bjc.1996.591

**Published:** 1996-11

**Authors:** T. G. Gantchev, N. Brasseur, J. E. van Lier

**Affiliations:** Department of Nuclear Medicine and Radiobiology, Faculty of Medicine, University of Sherbrooke, QC, Canada.

## Abstract

**Images:**


British Journal of Cancer (1996) 74, 1570-1577
© 1996 Stockton Press All rights reserved 0007-0920/96 $12.00

### Combination toxicity of etoposide (VP-16) and photosensitisation with a water-soluble aluminium phthalocyanine in K562 human leukaemic cells

TG Gantchev, N Brasseur and JE van Lier

*Department of Nuclear Medicine and Radiobiology, Faculty of Medicine, University of Sherbrooke, Sherbrooke, QC, J1H 5N4, Canada.*

**Summary** Etoposide (VP-16) is an anti-cancer drug commonly used against several types of tumours and leukaemia, either alone or in combination chemotherapy. Photodynamic therapy (PDT) is another, relatively new modality for treatment of various malignancies. The interactions between VP-16 and PDT, using aluminium tetrasulphophthalocyanine as photosensitiser, in K562 human leukaemic cells were investigated. Cell responses to individual and combined drug treatment under different experimental conditions revealed synergistic drug toxicity. The latter was evident from various events of cell response, including supra-additive accumulation of cells in $G_2$/M cell cycle phase and endonucleolytic DNA fragmentation (apoptosis). The involvement of the cellular antioxidant system in the synergistic interactions of photosensitisation and VP-16 is proposed.

**Keywords:** etoposide; phthalocyanine; photosensitisation; combination therapy; synergy

Etoposide[4′-dimethylepipodophyllotoxin-9-(4,6-*O*-ethylidene-β-D-glucopyranoside, VP-16] is an important antineoplastic agent used against several tumour types, either alone (Issel *et al.*, 1984), or in combination therapy (Aisner and Lee, 1991). Etoposide is also commonly used for the treatment of acute myelogenous leukaemia (Champlin and Gale, 1987). The cytotoxicity of VP-16 is generally believed to be based on introduction of DNA damage by drug interference with breakage-reunion reaction of DNA-topoisomerase II (formation of DNA-protein cross-links) (Glisson and Ross, 1987; Liu, 1989) and/or induction of direct DNA strand breaks and adducts (van Maanen *et al.*, 1988*a*, *b*; Mans *et al.*, 1991). In contrast to the topoisomerase II (topo II) poisoning by VP-16 itself, the direct inactivation of DNA was suggested to be conjugated with oxidation-reduction activation of VP-16 in the cellular environment (Mans *et al.*, 1990, 1992). In particular, cytochrome P450-dependent mono-oxygenases, peroxidases, prostaglandin synthetase, tyrosinase, etc. may be involved in VP-16 metabolic transformation (van Maanen *et al.*, 1987; Haim *et al.*, 1991; Gantchev *et al.*, 1994*a*). Evidence has been found that peroxidative metabolic products of VP-16, e.g. the *ortho*-quinone derivative of etoposide, as well as the short-lived intermediates (phenoxyl and semi-quinone free radicals) are involved in various oxidative reactions and DNA damage (Mans *et al.*, 1990, 1991; Sinha *et al.*, 1990). Recent studies suggest that the interactions of VP-16 free radicals with intracellular reductants (thiols, ascorbic acid, etc.) might play an essential role in the cytotoxic activity of the drug as well (Mans *et al.*, 1992; Kagan *et al.*, 1994; Yokomizo *et al.*, 1995).

Light activation of photosensitisers that have been accumulated in tumours is the basis of the photodynamic therapy (PDT) of cancer. Cytotoxic action of photosensitisers may involve oxidative damage to different cell constituents, including depletion of the pool of cell antioxidants (free and protein-bound thiols, ascorbic acid, α-tocopherol, etc.) (Buettner, 1984, Shopova and Gantchev, 1990, Gantchev and van Lier, 1995). Metallo-phthalocyanines (MePc) constitute a class of dyes proposed as second-generation photodynamic agents to supplant Photofrin, a mixture of porphyrin derivatives, currently used in the clinical treatment of various malignancies. Highly water-soluble phthalocyanines, such as di- through tetrasulphonated derivatives ($MePcS_{2-4}$) partially localise in cytoplasm and photosensitise intracellular generation of hydroxyl and organic free radicals (Gantchev *et al.*, 1994*b*). Among different effects of photosensitised cell damage, tetrasulphonated Al- and $ZnPcS_4$ have been shown to weaken cell viability by inactivation of catalase (Gantchev and van Lier, 1995) and inflicting damage to DNA (Hunting *et al.*, 1987; Gantchev *et al.*, 1994*c*). We have also shown that these phthalocyanines can initiate oxidative transformations of VP-16 in solution via photosensitised generation of VP-16 phenoxyl radical (Gantchev *et al.*, 1994*a*).

In view of the above properties of phthalocyanines and the interrelation between the rate of VP-16 oxidative transformation, its cytotoxicity, and the activity of intracellular antioxidant systems, we hypothesised that PDT, in conjunction with etoposide, could result in enhanced cytotoxicity. Analysis of different effects of combination therapy with VP-16 and $AlPcS_4$ photosensitisation against K562 chronic myelogenous leukaemia cells is the subject of the present work.

### Materials and methods

### *Chemicals*

Etoposide (VP-16) was purchased from Sigma (St Louis, MO, USA). The compound was dissolved in dimethyl sulphoxide (DMSO) at 5 mM, aliquoted and stored at −20°C. Further dilutions were made in RPMI medium immediately before use. Although the final DMSO concentrations of 1−2% in cell cultures were not toxic, all control groups received an equivalent amount of DMSO. Aluminium tetrasulphonated phthalocyanine ($AlPcS_4$) was synthesised in our laboratory, purified by high-performance liquid chromatography (HPLC) and dialysis to homogeneity and dissolved in phosphate-buffered saline (PBS) to yield a stock solution of 1.5−2.0 mM, as measured spectrophotometrically ($\varepsilon_{695\,nm} = 2.5 \times 10^4\ M^{-1}\ cm^{-1}$ in dimethylformamide). All other chemicals used were of the highest available purity.

### *Cell culture and drug treatments*

Human chronic myelogenous leukaemia cells, K562 (ATCC CCL 243), were grown in RPMI-1640 medium, supplemented with glutamine, 10% fetal bovine serum (FBS), 50 μg $ml^{-1}$ gentamycin and 10 mM Hepes. All experiments were

Correspondence: TG Gantchev
Received 28 March 1996; revised 4 June 1996; accepted 12 June 1996

performed with exponentially growing (asynchronous) cells. Usually 12–14 h before the experiment, the cells were seeded in a complete RPMI medium (3:1 v/v parts fresh to conditioned medium) at cell density of approximately $0.4 \times 10^6$ cells $ml^{-1}$. On the next day the cell density was adjusted by a small volume of fresh medium to give $0.5 \times 10^6$ cells $ml^{-1}$ and cells were exposed to drugs (VP-16 and $AlPcS_4$ alone, or mixed). All experiments were performed in triplicate flasks. In a typical protocol, after incubation with drugs, cells were first washed once with PBS, and a second time with RPMI (without FBS). After resuspending in a complete RPMI medium (conditioned to fresh medium = 1:3 v/v), cells were exposed to broad spectrum red light ($\lambda \geqslant 580$ nm; I = 10 mW $cm^{-2}$). Cell suspensions (12–15 ml) were exposed to red light in ventilated (green-cap Falcon, 75 $cm^{-2}$) incubation flasks and were gently shaken during irradiation. Cells were incubated at $5 \times 10^5$ cells $ml^{-1}$, or diluted to $5 \times 10^4$ cells $ml^{-1}$ to obtain a longer exponential phase. Control experiments performed on cells that were first photosensitised in PBS and then resuspended in culture medium did not show any significant difference in cell survival. Dark incubation with $AlPcS_4$ was not cytotoxic. Exposure to red light of cells incubated with VP-16, in the absence of $AlPcS_4$, did not result in altered VP-16 toxicity.

### *Cytotoxicity assays and drug interaction analysis*

Cell growth inhibition was monitored by means of dye exclusion (staining with 0.4% Trypan blue) and/or by Coulter counter measurements. Under our experimental conditions, the doubling time of control cells was 22–24 h. The growth suppression activity of drugs was compared by estimation of the exponential rate constants using initial portions of growth curves (e.g. 48–60 h after treatment). Clonogenic assay was performed in a standard fashion by plating cells in soft (0.33%) agar in 35 mm Petri dishes (usually 300, 600 and 1200 cells per dish in triplicate).

To analyse the dose–effect relationships in combination treatment of cells with VP-16 and $AlPcS_4$ photosensitisation, two algorithms were used. A simple estimate was performed using the fractional product method (Veleriote and Liu, 1975). This 'multiplicative' model predicts additivity, antagonism and/or synergism of two drugs based on the comparison between the individual drug effect on cell survival, $f_u$ (expected) = $(f_u)_1 \times (f_u)_2$ and the experimentally obtained value of cell survival after combination treatment, $(f_u)_{1,2}$. The symbol $(f_u)_{1,2}$ stands for 'fraction unaffected' and is equal to $1-(f_a)_{1,2}$, where $f_a$ ('fraction affected') refers to the fraction of cells responding to various concentrations of the two drugs in combination. By definition, $(f_u)_{1,2}$ lower, equal to or larger than $f_u$ (expected) determines the border lines of synergistic (supra-additive), additive and antagonistic drug interactions respectively. In a specially designed set of experiments, we also performed a detailed analysis of drug interactions using the median effect principle. Unlike other methods often used to predict drug interactions in biological systems (usually applicable only to mutually exclusive interactions), the median effect principle may be used to analyse both mutually exclusive and mutually non-exclusive interactions (Chou and Talalay, 1983). We used the linearised median effect equation in the form of $\log [(f_u)^{-1}-1] = m \log (D) - m \log (D_m)$, where 'm' is the Hill-type coefficient determining the sigmoidality of the dose–effect curve; 'D' is drug(s) dose; and $D_m = IC_{50}$ is the dose required to produce the median effect. The combination index (CI) was calculated at $f_a$ levels of 0.1 intervals, as described by Chou and Talalay (1983). According to this algorithm, CI values <1.0 indicate synergism; CI >1.0, antagonism; and CI approximately 1.0, additivity.

### *Flow cytometric cell cycle analysis*

After given time periods following drug removal, cells were washed and resuspended in 200 $\mu$l PBS containing 5.5 mM glucose and 1 mM EDTA. While vortexing, 1 ml of cold 70% ethanol was added and cells were incubated at 4°C for 30 min. Thereafter, the cells were transferred to PBS–glucose–EDTA buffer containing 0.5% Tween-20 and incubated for 1 h at 4°C to rehydrate. Following a brief exposure to RNAase, DNA was stained with 50 $\mu$g $ml^{-1}$ propidium iodide. Before cytoflow analysis, cells were filtered through nylon mesh to avoid clumps. The readings were performed on a Hewlett-Packard computer-controlled Becton Dickinson FACScan instrument. DNA chromatograms were analysed by the SOBR software supplied by the manufacturer. In cases in which high levels of cell debris were present, appropriate gate cut-off settings were used.

### *Analysis of internucleosomal DNA fragmentation*

The integrity of DNA from drug-treated and control cells was assessed by agarose electrophoresis. After removal of culture medium at different post-treatment times, cells ($0.5 \div 2 \times 10^6$) were resuspended in 150 $\mu$l PBS–glucose–EDTA buffer and transferred to Eppendorf microfuge tubes. Immediately after that, 0.5 ml of lysis buffer [0.8% sodium dodecyl sulphate (SDS) and 100 mM EDTA in 50 mM Tris-HCl, pH = 7.4] was added and cells were incubated at room temperature for 20 min. After addition of 125 $\mu$l of 5 M sodium chloride to each tube, samples were gently mixed and left overnight at 4°C. On the next day, samples were microcentrifuged at 12 000 × *g* for 30 min at 4°C and the pellets were carefully removed and discarded. Supernatants were further incubated with 0.45 mg $ml^{-1}$ proteinase K for 2 h at 50°C. After addition of 50 $\mu$l of 5 M sodium chloride and 1 ml isopropanol, samples were left for 1 h at −20°C and microfuged. The pellet was washed with 70% ethanol and dried under vacuum. Resuspended in 20 $\mu$l TE buffer, pellets were incubated with 0.8 mg $ml^{-1}$ RNAase A at 37°C for 3 h. After mixing with 4 $\mu$l of 6 × loading buffer (TE/bromophenol/glycerol), samples were transferred onto 1.2% agarose gel and electrophoresed at 40 V for 4 h. Gels were stained for 40 min with 10 ng $ml^{-1}$ ethidium bromide, destained for 30–60 min in distilled water and photographed under UV light. Series of experiments were performed to selected post-treatment time periods and number of cells required to give early and easily detectable DNA fragmentation in combination with selected multiple drug dose equivalents to reveal drug interaction.

### Results

### *Cell growth and loss of clonogenicity*

To determine the toxicity range of individual drugs and their combination effects, several parameters were examined: incubation times of cells with drugs, drug concentrations and the applied light dose for $AlPcS_4$ activation. The cellular response to drug treatment was followed by monitoring the growth rate and cell clonogenic activity. Growth curves shown in Figure 1 exemplify the evolution of immediate drug toxicity with respect to drug incubation time, concentrations and light dose. For individual drugs and their combinations, growth curves show a proliferation lag period, followed either by predominant cell regrowth or cell death. At low levels of drug treatment (Figure 1a and b), and during the initial proliferation lag-times (e.g. at post-treatment times shorter than 24 h), the number of dead cells did not exceed 5–8% of the total, but cell cycle progression was largely inhibited (see below). Figure 2 demonstrates the effect of etoposide concentration on clonogenic activity of K562 cells, either when the drug was applied alone or in combination with $AlPcS_4$ photosensitisation. Under the employed incubation conditions of cells with VP-16 (20–60 min), the $IC_{50}$ concentration of the drug, as determined from cloning experiments, was in the range from 15 to 6 $\mu$M, and was markedly decreased after combined treatment (Figure 2). The $IC_{50}$ of $AlPcS_4$ varied with preirradiation incubation time and light dose (D), and, for example, after 4 h preincubation was

**Figure 1** Growth of K562 cells incubated for different times with VP-16 (●); AlPcS$_4$ (▲); or both (◆) and exposed to red light. (**a**) 4 $\mu$M AlPcS$_4$ and 2 $\mu$M VP-16 for 1 h, light dose (D) = 7.2 J cm$^{-2}$; (**b**) 30 $\mu$M AlPcS$_4$ for 5 h and 7.5 $\mu$M VP-16 for 1 h, D = 6 J cm$^{-2}$; (**c**) 20 $\mu$M AlPcS$_4$ for 5 h and 10 $\mu$M VP-16 for 1 h, D = 9 J cm$^{-2}$; and (**d**) 40 $\mu$M AlPcS$_4$ for 5 h and 30 $\mu$M VP-16 for 2 h, D = 9 J cm$^{-2}$. Control cells (○). Incubation time starts after drug removal and light exposure. Averaged data from triplicate experiments.

about 20 $\mu$M (D = 9 J cm$^{-2}$), and increased when cells were exposed to photosensitiser for a shorter time (e.g. 30–35 $\mu$M at 2 h). The simple 'multiplicative' model and data from cloning experiments (e.g. shown in Figure 2) largely predict supra-additive (synergistic) combined toxicity [i.e. $(f_u)_{1,2} < f_u$ (expected) $= (f_u)_1 \times (f_u)_2$, Materials and methods]. A complete analysis of drug interaction, however, was performed using the median effect principle, and is described below.

*Cell cycle arrest*

It has been shown previously that continuous exposure to low concentrations of VP-16 allows cells of different origin to traverse the cell cycle until a predominant number of them are blocked with the DNA content of $G_2$ cells, but cannot progress to the mitotic stage (Krishan *et al.*, 1975). In the present study, the cells were incubated with VP-16 for given time periods, and, thereafter, the drug was removed. Under conditions of low drug load ($\leqslant IC_{50}$), VP-16 induced transient $G_2$/M arrest of K562 cells (Figure 3). Cell size analysis performed by means of Coulter counter measurements showed a parallel and transient increase in cell volume, obviously associated with arrest in $G_2$-phase. Higher doses and longer exposures to the drug, however, resulted in S- and mixed S/$G_2$-phase arrest (not shown). It is noteworthy that in this case, and after longer post-treatment incubation times

**Figure 2** Clonogenic survival after exposure of K562 cells to different concentrations of VP-16 for 1 h (△); and after combination treatment when cells were preincubated with 4 $\mu$M AlPcS$_4$ for 20 h (○); or with 20 $\mu$M AlPcS$_4$ for 4 h (●). Light dose in both cases, (D) = 7.2 J cm$^{-2}$. Averaged data from triplicate experiments.

**Figure 3** Effects after exposure to VP-16 and $AlPcS_4$ on cell cycle distribution of K562 cells. (**1b**) DNA chromatograms obtained from cells 26 h after treatment with 20 $\mu$M $AlPcS_4$ for 5 h; (**1c**) 10 $\mu$M VP-16 for 1 h; and (**1d**) combination of **1b** and **1c**. Light dose (D) = 9 J cm$^{-2}$. (**2b**) Chromatograms obtained 20 h after cell treatment with 4 $\mu$M $AlPcS_4$ for 1 h; (**2c**) 2 $\mu$M VP-16 for 1 h; and (**2d**) combination of **2b** and **2c**. Light dose 7.2 J cm$^{-2}$. **1a** and **2a** show the chromatograms of control cells.

(>24 h), cell cycle analysis was more complicated owing to the presence of high amounts of dead cells and a cell population with quadraploid DNA content. In contrast, $AlPcS_4$ photosensitisation under a wide range of light doses and preillumination incubation times (2–24 h), induced only $G_2$/M arrest. When cells were treated with doses around the $IC_{50}$ values of both drugs, the maximum number of $G_2$/M-arrested cells was reached after 15–20 h post-treatment incubation and did not exceed a value of approximately 60% of the total (Figure 4a). The cell accumulation in $G_2$/M was accompanied by a depletion, mainly of S-phase population, with very little variation in the $G_1$-phase content. During combined treatment, the cells were also arrested in $G_2$/M (Figure 3). The extent of cell response after combined treatment varied and depended on the individual drug effect. Thus, in conditions when individual drugs were relatively more toxic (e.g. at doses approximately equal to the $IC_{50}$, see also Figure 1b) and the maximum fraction of cells arrested by individual drug treatment was close to 60%, there was no significant increase in the $G_2$/M population after combined treatment. However, under these conditions, the maximum number of $G_2$/M-arrested cells after combined treatment was reached at later times and the total proliferation block lasted longer (Figure 4a). It is noteworthy that in these conditions, during the growth lag period and after that, the number of dead cells remained relatively high, as also seen in DNA chromatograms exhibiting a higher percentage of cells with abnormal DNA content (debris and, perhaps, apoptotic cells). In contrast, when the toxic effects of individual drugs were low (growth curve shown in Figure 1a), combined treatment induced a supra-additive (synergistic) accumulation of cells in $G_2$/M-phase, as also proved by the 'multiplicative' model (Figure 4b). Synergistic increase of the number of cells accumulated in $G_2$/M-phase was reached under various other conditions, but especially when the toxicity of at least one of the drugs was kept low (Figure 4c).

### *Internucleosomal DNA degradation*

K562 leukaemic cells are known to be relatively resistant to VP-16-induced internucleosomal DNA fragmentation (apoptosis) (Dubrez *et al.*, 1995). Thus, exposure of cells to VP-16 of concentrations around or higher than $IC_{50}$ (5 $\mu$M and 10 $\mu$M for 1 or 2 h) did not reveal any significant DNA fragmentation during post-treatment incubation times up to 36 h, as assessed by agarose gel electrophoresis (Figure 5, lanes 4 and 5). DNA fragmentation was induced when cells were treated with higher VP-16 concentrations (e.g. $5 \times IC_{50}$), but the pattern was obscured even when DNA was isolated from $2 \times 10^6$ or more cells (not shown). In contrast, $AlPcS_4$ photosensitisation was a much more potent inducer of DNA fragmentation (apoptosis). Internucleosomal DNA damage was readily detected 4–6 h post treatment in DNA samples isolated from $0.5-1.0 \times 10^6$ cells treated with drug dose $< IC_{50}$ (Figure 5, lane 3). This pattern was persistent and progressively augmented during the next 48–72 h of post-treatment incubation. Figure 5 also shows the results from a typical experiment when cells were exposed to two drug concentrations individually, or to their combination. Under the specified conditions (see figure legend), no extensive DNA laddering took place after treatment with either 5 $\mu$M $AlPcS_4$ or 5 and 10 $\mu$M VP-16 alone (lanes 2, 4 and 5). Internucleosomal cleavage, however, was well pronounced in all samples when cells were subjected to combined treatment. It is also noteworthy that in every case when DNA internucleosomal fragments were easily seen, they were superimposed on DNA smears. This is probably caused by the occurrence of two parallel processes of DNA degradation when cells were more heavily damaged: unprogrammed (necrosis) and programmed (apoptosis) cell death. The technique of agarose electrophoresis, as applied in this study, does not permit a direct quantitative estimate of the levels of internucleosomal damage induced by single and combined treatment. From the selected drug doses and the results shown in Figure 5, however, it is evident that simultaneous employment of VP-16 with photodynamic treatment induces supra-additive increase of DNA fragmentation.

### *Median effect – combination index analysis*

The results shown in previous sections indicate that drug combination treatment in most experimental conditions is supra-additive (synergistic). To evaluate modes of drug interactions, we designed experiments suitable for application of combination index analysis. This analysis is based on the median effect principle and is a statistical technique that allows formal evaluation of the nature of interaction between two cytotoxic agents. Cells were incubated with different concentrations of drugs, but at constant molar ratio (VP-16/$AlPcS_4$ = 1/2). Before irradiation, the cells were exposed to $AlPcS_4$ for 1.5 h, followed by co-incubation with VP-16 for 0.5 h. Thereafter, the cells were washed and irradiated ($D = 7.2$ J $cm^{-2}$). Drug toxicity was assessed by their effect on growth rate and clonogenicity. Figure 6 demonstrates that, under these conditions, the $AlPcS_4$ phototoxicity was low, as estimated by both growth rate inhibition and loss of clonogenicity. However, growth rate assessment of VP-16 toxicity underestimates the reproductive toxicity of the drug (Figure 6). The median effect plots derived from cloning experiments and linear regression data fit are shown in Figure 7. The dose–effect relationships of the individual drugs are

**Figure 4** Time-course of the accumulation of cells in $G_2$/M phase after treatment with (**a**) 20 $\mu$M $AlPcS_4$ for 5 h (▲); 10 $\mu$M VP-16 for 1 h (●); and their combination (◆). Control cells (○). After treatment of cells with (**b**) 4 $\mu$M $AlPcS_4$ for 1 h; 2 $\mu$M VP-16 for 1 h; and their combination; (**c**) 4 $\mu$M $AlPcS_4$ for 20 h; 2 $\mu$M VP-16 for 1 h; and their combination. Light doses: (**a**) $D = 9$ J $cm^{-2}$, and (**b** and **c**) $D = 7.2$ J $cm^{-2}$. Averaged data from triplicate experiments.

**Figure 5** Internucleosomal DNA fragmentation in K562 cells 4 h after treatment with drugs. Lanes: (1) control; (2 and 3) individual drug treatment with 5 $\mu$M and 10 $\mu$M $AlPcS_4$ for 4 h; (4 and 5) 5 $\mu$M and 10 $\mu$M VP-16 for 1 h; (6 and 7) combination treatments: 5 $\mu$M $AlPcS_4$ and 5 $\mu$M or 10 $\mu$M VP-16; (8 and 9) 10 $\mu$M $AlPcS_4$ and 5 $\mu$M or 10 $\mu$M VP-16. Lane 10, marker DNA (1, 2 and 3 kb fragments). Photosensitisation light dose, (D) = 7.2 J $cm^{-2}$. DNA was isolated from $0.5 \times 10^6$ cells in each case.

**Figure 6** Cell survival (loss of clonogenicity, open symbols) and growth rate inhibition (closed symbols) after photosensitisation with $AlPcS_4$ (△, ▲), incubation with VP-16 (○, ●) and combination treatment (◇, ◆). Cells were incubated with various concentrations of $AlPcS_4$ for 2 h and with VP-16 for 30 min. Drug concentration ratio VP-16/$AlPcS_4$ = 1/2. Light dose, (D) = 7.2 J $cm^{-2}$. Combination treatment curves are plotted as a function of VP-16 concentration. Growth inhibition was estimated by comparison of growth rate constants. Averaged data from triplicate samples.

strictly linear ($r \geqslant 0.999$, lines 1 and 2), while the plot obtained for the mixture of the two drugs tends to concave upward ($r = 0.99$) and intersects the plot of the more active drug (Figure 7, line 3). It is, therefore, apparent that the combined drug action is synergistic. This was further confirmed by calculating the combination index values (CI) for a wide range of $f_a$ ('fraction affected', Figure 8). Since the plots for the individual drugs are almost parallel (lines 1 and 2 in Figure 7), and the plot for the combined treatment (line 3) intersects the plot of the more active drug, it is likely that the two drugs interact as mutually non-exclusive. The combination index was calculated assuming both possibilities, mutually exclusive and non-exclusive interactions. The plots shown in Figure 8 indicate that these two models predict synergistic toxicity ($CI \leqslant 1$) under conditions in which combined treatment results in $f_a \geqslant 0.10$ or $f_a \geqslant 0.15$ respectively. For a comparison, CI values obtained from growth inhibition data were also calculated and included in Figure 8. It can be seen that synergism in the latter case is somewhat overestimated, probably due to the underestimation of VP-16 toxicity by this criterion.

### Discussion

We have investigated the effects of individual toxicity of etoposide (VP-16) and photodynamic treatment with $AlPcS_4$, as well as the efficacy of drug combination against K562 human leukaemic cells. Similarly, in our previous studies with Namalva Burkitt's lymphoma cells (Gantchev *et al*., 1994*b*, photosensitisation of K562 cells results in division block and, depending on the extent of the treatment, is followed either by cell regrowth or by predominant cell death. Unlike photosensitisation with Photofrin, which induces S-phase cell cycle arrest (Gantchev *et al*., 1994*d*), in the present study we show that $AlPcS_4$ photosensitisation arrests cells in $G_2$/M-phase. Under the conditions of relatively mild photodynamic treatment employed, no evidence for immediate membrane disintegration was found. In contrast, most of the toxic effects were delayed and emerged as a function of post-treatment incubation time, as seen, for example, by the time course of the accumulation of $G_2$/M-arrested cells and internucleosomal DNA cleavage. Similar cell growth inhibition effects were observed after short (0.5–2 h) exposure of

**Figure 7** (1) Median-effect plots for the cytotoxicity of VP-16; (2) $AlPcS_4$ and (3) their combination. Experimental data from clonogenic survival (Figure 6).

**Figure 8** Plots of combination index (CI) *vs* fraction affected ($f_a$) for $[VP\text{-}16]/[AlPcS_4] = 1/2$, data from clonogenic toxicity of the drugs (■, ●), or growth inhibition (□, ○), as calculated for mutually exclusive (○, ●) and mutually non-exclusive drugs (□, ■).

K562 cells to VP-16. The cytotoxicity of $AlPcS_4$-PDT, however, evolved faster compared with VP-16. In particular, PDT-induced growth and clonogenic inhibition developed at similar rates, while VP-16 displayed a lower level of growth suppression, compared with the more effective clonogenic inhibition (Figure 6). When applied alone, VP-16 did not induce any significant internucleosomal DNA degradation (apoptosis). Apoptotic cell death was an early response to $AlPcS_4$-PDT and was augmented after combined drug treatment (Figure 5). Synergistic cell response to combined drug treatment was attained under various conditions, as clearly demonstrated by growth inhibition rates (Figure 1a and d), loss of clonogenicity (Figure 2) and accumulation of cells in $G_2$/M cell cycle phase (Figure 3b and c). In particular, synergism (supra-additivity) in cell cycle arrest took place when the fraction of $G_2$/M cells arrested by single drug treatment did not exceed a value of approximately 60%. Quantitatively, this value corresponds to the normal S-phase fraction of exponentially growing K562 cells and implies that drug treatment exclusively affects cells during the stage of DNA synthesis. After combined drug treatment and during the accumulation of cells in $G_2$/M-phase, a progressive increase of DNA internucleosomal fragmentation was observed. It is, therefore, likely that when cells are more extensively damaged by combined drug treatment, $G_2$/M-arrested cells tend to fall out from the cell cycle and die (eventually by apoptosis). The molecular mechanisms that are involved in $AlPcS_4$-PDT-induced apoptosis were not addressed in this study. However, we suggest that they do not encompass the phospholipases C and A2, signalling pathway shown to operate during photosensitisation with cytoplasmic membrane-localising (unsubstituted) AlPc (Agarwal *et al.*, 1993). In the latter case, the primary target is the cytoplasmic membrane, and it has been shown that DNA fragmentation evolves within minutes after treatment, in contrast to the slower accumulation of apoptotic cells after $AlPcS_4$ and/or VP-16 treatment. Water-soluble phthalocyanines, such as $AlPcS_4$, have been implicated in the induction of DNA strand breaks, DNA–protein cross-links (Hunting *et al.*, 1987; Gantchev *et al.*, 1994*c*), and impairment of cell organelles, including peroxisomes (Peng *et al.*, 1991). All these oxidative processes may account for $AlPcS_4$ cytotoxicity and represent primary events in $AlPcS_4$ - PDT-mediated internucleosomal DNA cleavage.

The cytostatic/cytotoxic action of VP-16 is generally believed to involve formation of DNA–protein cross-links and breaks. However, it is likely that drug toxicity is not limited to topoisomerase II (topo II) poisoning only. Thus, a recent study argued on the lack of correlation between the level of DNA–protein cross-links and cytotoxicity in several leukaemic cell lines (Dubrez *et al.*, 1995). Previously, other studies have also indicated that the interaction of VP-16 with topo II and cytotoxicity of the drug can be uncoupled, e.g. drug interaction with topo II might be not intrinsically cytotoxic, and therefore it has been suggested that VP-16 cytotoxic action might involve one, or more, intervening metabolic steps (Kaufmann, 1989; Walker *et al.*, 1991). Oxidoreductive transformations of VP-16 induced by a variety of intracellular enzymatic systems have been shown to constitute a pathway for modulation of drug activity (van Maanen *et al.*, 1987; Mans *et al.*, 1990; Haim *et al.*, 1991). Furthermore, the intermediate species from VP-16 metabolism: VP-16 phenoxyl and semi-quinone radicals, and one of the final products of drug demethylation (VP-16 *ortho*-quinone) have been implicated as important cytotoxic agents (Mans *et al.*, 1991, 1992; Ritov *et al.*, 1995). It is, however, noteworthy that the intracellular accumulation of these products depends strongly on the presence of cellular antioxidants (e.g. reduced ascorbate and thiols) (Gantchev *et al.*, 1994*a*; Kagan *et al.*, 1995).

The combination index analysis of $AlPcS_4$ photosensitisation and VP-16 treatment as performed in the present work largely predicts synergistic toxicity of the two drugs. The experiments throughout this study were performed so that effects, such as cell synchronisation, were avoided and cannot account for the observed synergy. Moreover, the median effect plot of combined toxicity (Figure 7) suggests that the drugs are likely to interact as mutually non-exclusive. Non-exclusivity of drug interaction would imply that $AlPcS_4$ and VP-16 share a common intracellular target and/or interact chemically. It is not known if $AlPcS_4$ photosensitisation can directly interfere with the topo II/DNA breakage–reunion process, but it is recognised that photosensitisation (Hunting *et al.*, 1987; Gantchev *et al.*, 1994*c*) and VP-16 metabolites (van Maanen *et al.*, 1988*b*; Mans *et al.*, 1990, 1991; Sinha *et al.*, 1990) can instantly induce damage to DNA. Therefore, based on the assumption that metabolic oxidoreductive transformations of VP-16 are important for its cytotoxic action, we suggest that photosensitisation-induced depletion of intracellular reductants facilitates VP-16 metabolism, probably on the level of VP-16 phenoxyl free radical transformations. Generation of the VP-16 phenoxyl radical is the primary step in the enzymatic metabolism of the drug and the interactions of the phenoxyl radical with cell antioxidants prevents etoposide from further transformations and results in recovery of its original form. Also, the VP-16 phenoxyl radical may itself initiate oxidative cell damage (Ritov *et al.*, 1995). A parallel drug interaction mechanism may involve direct $AlPcS_4$-mediated photo-oxidation of VP-16 to yield the phenoxyl radical (Gantchev *et al.*, 1994*a*). Complete elucidation of the role of these different pathways and the involvement of additional processes that may explain the observed synergistic drug toxicity will require further experimental work. It is relevant to note that a recent study showed synergism in cells subjected to gamma radiation and etoposide treatment (Haddock *et al.*, 1995). Since the oxidative processes induced by PDT and radiotherapy are similar, it is possible that the synergistic interactions with etoposide are based on the same mechanisms. Although the presented results only involve *in vitro* synergy between photodynamic treatment and etoposide toxicity, our findings may provide alternative perspectives for clinical PDT and etoposide applications.

**Acknowledgement**

TGG acknowledges support from the Ministère de l'Enseignement Supérieur et de la Science du Gouvernement du Québec (Programme d'Actions Structurantes).

### References

AGARWAL ML, LARKIN HE, ZAIDI SIA, MUKHTAR H AND OLEINICK NL. (1993). Phospholipase activation triggers apoptosis in photosensitized mouse lymphoma cells. *Cancer Res.*, **53**, 5897-5902.

AISNER J AND LEE EJ. (1991). Etoposide. Current and future status. *Cancer*, **67**, (suppl. 1), 215-219.

BUETTNER GR. (1984). Thiyl radical production with hematoporphyrin derivative, cytsteine and light: a spin trapping study. *FEBS Lett.*, **177**, 295-299.

CHAMPLIN R AND GALE RP. (1987). Acute myelogenous leukemia: recent advances in therapy. *Blood*, **69**, 1551-1562.

CHOU T-C AND TALALAY P. (1983). Quantitative analysis of dose-effect relationships: the combined effects of multiple drugs or enzyme inhibitors. In *Advances in Enzyme Regulation*, Vol. 22, Weber G (ed.), pp. 27-55. Pergamon Press: Oxford.

DUBREZ L, GOLDWASSER F, GENNE P, POMMIER Y AND SOLARY E. (1995). The role of cell cycle regulation and apoptosis triggering in determining the sensitivity of leukemic cells to topoisomerase I and II inhibitors. *Leukemia*, **9**, 1013-1024.

GANTCHEV TG AND VAN LIER JE. (1995). Catalase inactivation following photosensitization with tetrasulfonated metallophthalocyanines. *Photochem. Photobiol.*, **62**, 123-134.

GANTCHEV TG, VAN LIER JE, STOYANOVSKY DA, YALOWICH JC AND KAGAN VE. (1994*a*). Interactions of phenoxyl radical of antitumour drug, etoposide, with reductants in solution and in cell and nuclear homogenates: electron spin resonance and high-performance liquid chromatography. *Methods Enzymol.*, **234**, 631-642.

GANTCHEV TG, URUMOV IJ, HUNTING DJ AND VAN LIER JE. (1994*b*). Photocytotoxicity and intracellular generation of free radicals by tetrasulphonated A1- and Zn-phthalocyanines. *Int. J. Radiat. Biol.*, **65**, 289-298.

GANTCHEV TG, GOWANS BJ, HUNTING DJ, WAGNER JR AND VAN LIER JE. (1994*c*). DNA strand scission and base release photosensitized by metallo-phthalocyanines. *Int. J. Radiat. Biol.*, **66**, 705-716.

GANTCHEV TG, URUMOV IJ AND VAN LIER JE. (1994*d*). On the relationship between rate of uptake of photofrin and cellular responses to photodynamic treatment *in vitro*. *Cancer Biochem. Biophys.*, **14**, 23-34.

GLISSON BS AND ROSS WE. (1987). DNA topoisomerase II: a primer on the enzyme and its unique role as a multidrug target in cancer chemotherapy. *Pharmacol. Ther.*, **32**, 89-106.

HADDOCK MG, AMES MM AND BONNER JA. (1995). Assessing the interaction of irradiation with etoposide or idarubicin. *Mayo Clin. Proc.*, **70**, 1053-1060.

HAIM N, NEMEC J, ROMAN J AND SINHA B. (1991). Peroxidase-catalyzed metabolism of etoposide (VP-16-213) and covalent binding of reactive intermediates to cellular macromolecules. *Cancer Res.*, **47**, 5835-5840.

HUNTING DJ, GOWANS BJ, BRASSEUR N AND VAN LIER JE. (1987). DNA damage and repair following treatment of V-79 cells with tetrasulfonated phthalocyanines. *Photochem. Photobiol.*, **45**, 769-773.

ISSEL BF, MUGGIA FM AND CARTER SK. (1984). *Etoposide (VP-16). Current Status and New Developments*. Academic Press: Orlando, U.S.A.

KAGAN VE, YALOWICH JC, DAY BW, GOLDMAN R, GANTCHEV TG AND STOYANOVSKY DA. (1994). Ascorbate is the primary reductant of the phenoxyl radical of etoposide in the presence of thiols both in cell homogenates and in model systems. *Biochemistry*, **33**, 9651-9660.

KAUFMANN SH. (1989). Induction of endonucleolytic DNA cleavage in human acute myelogenous leukemia cells by etoposide, camptothecin, and other cytotoxic anticancer drugs: a cautionary note. *Cancer Res.*, **49**, 5870-5878.

KRISHAN A, PAIKA K AND FREI, III, E. (1975). Cytofluorometric studies on the action of podophyllotoxin and epipodophyllotoxins (VM-26, VP-16-213) on the cell cycle traverse of human lymphoblasts. *J. Cell Biol.*, **66**, 521-530.

LIU LF. (1989). DNA topoisomerase poisons as antitumour drugs. *Annu. Rev. Biochem.*, **58**, 351-375.

MANS DRA, RETÈL J, VAN MAANEN JMS, LAFLEUR MVM, VAN SCHAIK MA, PINEDO HM AND LANKELMA J. (1990). Role of the semi-quinone free radical of the anti-tumour agent etoposide (VP-16-213) in the inactivation of single- and double-stranded ΦX174 DNA. *Br. J. Cancer*, **62**, 54-60.

MANS DRA, LAFLEUR MVM, WESTMIJZE EJ, VAN MAANEN JMS, VAN SCHAIK MA, LANKELMA J AND RETÈL J. (1991). Formation of different reaction products with single- and double-stranded DNA by the *ortho*-quinone and the semi-quinone free radical of etoposide (VP-16-213). *Biochem. Pharmacol.*, **42**, 2131-2139.

MANS DRA, LAFLEUR MVM, WESTMIJZE EJ, HORN IR, BETS D, SCHUURHUIS GJ, LANKELMA J AND RETÈL J. (1992). Reactions of glutathione with the catechol, the *ortho*-quinone and the semi-quinone free radical of etoposide. *Biochem. Pharmacol.*, **43**, 1761-1768.

PENG Q, FARRANTS GW, MADSLIEN K, BOMMER JC, MOAN J, DANIELSEN HE AND NESLAND JM. (1991). Subcellular localization, redistribution and photoblenching of sulfonated aluminium phthalocyanines in human melanoma cell line. *Int. J. Cancer*, **49**, 290-295.

RITOV VB, GOLDMAN R, STOYANOVSKY DA, MENSHIKOVA EV AND KAGAN VE. (1995). Antioxidant paradoxes of phenolic compounds: peroxyl radical scavenger and lipid antioxidant, etoposide (VP-16), inhibits sarcoplasmic reticulum $Ca^{2+}$-ATPase via thiol oxidation by its phenoxyl radical. *Arch. Biochem. Biophys.*, **321**, 140-152.

SHOPOVA M AND GANTCHEV T. (1990). Comparison of the photosensitizing efficiencies of haematoporphyrin (HP) and its derivative (HPD) with that of free-base tetrasulphophthalocyanine (TSPC-$H_2$) in homogeneous and microheterogeneous media. *J. Photochem. Photobiol., B: Biology*, **6**, 49-59.

SINHA BK, ANTHOLINE WM, KALYANARAMAN B AND ELIOT HM. (1990). Copper ion-dependent oxy-radical mediated DNA damage from dihydroxy derivative of etoposide. *Biochim. Biophys. Acta*, **1096**, 81-83.

VALERIOTE F AND LIU H-S. (1975). Synergistic interaction of anticancer agents: a cellular perspective. *Cancer Chemother. Res.*, **59**, 895-900.

VAN MAANEN JMS, DE VRIES J, PAPPIE D, VAN DEN AKKER E, LAFLEUR MVM, RETÈL J, VAN DER GREEF J AND PINEDO HM. (1987). Cytochrome P-450-mediated *O*-demethylation: a route in the metabolic activation of etoposide (VP-16-213). *Cancer Res.*, **47**, 4658-4662.

VAN MAANEN JMS, RETÈL J, DE VRIES J AND PINEDO HM. (1988*a*). Mechanism of action of antitumor drug etoposide: a review. *J. Natl Cancer Inst.*, **80**, 1526-1533.

VAN MAANEN JMS, LAFLEUR MVM, MANS DRA, VAN DEN AKKER E, DE RUITER C, KOOTSTRA PR, PAPPIE D, DE VRIES J, RETÈL J AND PINEDO HM. (1988*b*). Effects of the ortho-quinone and catechol of the antitumor drug VP-16-213 on the biological activity of single-stranded and double-stranded ΦX174 DNA. *Biochem. Pharmacol.*, **37**, 3579-3589.

WALKER PR, SMITH C, YOUDALE T, LEBLANC J, WHITFIELD JF AND SIKORSKA M. (1991). Topoisomerase II-reactive chemotherapeutic drugs induce apoptosis in thymocytes. *Cancer Res.*, **51**, 1078-1085.

YOKOMIZO A, ONO M, NANRI H, MAKINO Y, OHGA T, WADA M, OKAMOTO T, YODOI J, KUWANO M AND KOHNO K. (1995). Cellular levels of thioredoxin associated with drug sensitivity to cisplatin, mitomycin C, doxorubicin, and etoposide. *Cancer Res.*, **55**, 4293-4296.